# Behavior Change Techniques for the Maintenance of Physical Activity in Cancer

**DOI:** 10.2196/53602

**Published:** 2023-11-28

**Authors:** Lara Edbrooke, Tamara L Jones

**Affiliations:** 1 The University of Melbourne Melbourne Australia; 2 The Peter MacCallum Cancer Centre Melbourne Australia

**Keywords:** cancer, physical activity, behavior change

## Abstract

Ester et al report the findings from a 2-arm cluster randomized controlled trial nested within a hybrid effectiveness-implementation study, which involved a 12-week exercise and behavior change program for rural and remote Canadians (Exercise for Cancer to Enhance Living Well [EXCEL]). The addition of 23 weeks of app-based physical activity monitoring to the EXCEL program did not result in significant between-group differences in physical activity at 6 months. While several behavior change techniques were included in the initial 12-week intervention, additional techniques were embedded within the mobile app. However, there is currently a lack of evidence regarding how many and which behavior change techniques are the most effective for people with cancer and if these differ based on individual characteristics. Potentially, the use of the mobile app was not required in addition to the behavior change support delivered to both groups as part of the EXCEL program. Further research should involve participants who may be in most need of behavioral support, for example, those with lower levels of self-efficacy. Suggestions for future research to tailor behavior change support for people with cancer are discussed.

## Introduction

Ester et al [1] recently report findings from a 2-arm cluster randomized controlled trial (RCT) nested within a hybrid effectiveness-implementation study providing rural and remote Canadians access to a 12-week exercise and behavior change program (Exercise for Cancer to Enhance Living Well [EXCEL]). Maintenance of physical activity (PA) behaviors beyond the completion of an exercise program is challenging for both healthy individuals and those with chronic health conditions including cancer. This is a critical issue, given the aging population and the known health benefits of increased PA, including management of common comorbidities such as type 2 diabetes and cardiac disease, prevention of secondary cancers, reduced risk of cancer recurrence, and improvements in survival [2]. Nonetheless, <25% of RCTs of exercise in cancer report significant between-group differences in PA when measured a minimum of 6 months following program completion [3]. The findings of this cluster RCT are similar [1]. Furthermore, in the intervention group, the addition of 23 weeks of app-based PA monitoring to the program did not cause significant between-group differences in self-reported moderate to vigorous physical activity at 6 months. Notably, each group reported PA levels below the recommended guidelines at baseline. Both groups reported significant increases in PA, with weekly increases in moderate to vigorous physical activity minutes (intervention vs control groups: baseline, 60.0, IQR 0.0-180.0 vs 40.0, IQR 0.0-135.0; week 12, 240.0, IQR 117.5-387.75 vs 225.0, IQR 102.5-352.5; week 24, 205.0, IQR 87.5-330.0 vs 160.0, IQR 55.0-180.0). The authors should be commended for their focus on recruiting rural and remote participants and longer-term follow-up, which are elements that are often lacking in evaluations of exercise program effectiveness [4].

## What Is Required to Change PA Behaviors?

In the initial 12-week component, both groups received the EXCEL “exercise and educate” program, which involved 5 educational topics delivered on the web and targeted the following behavior change techniques (BCTs): instruction on how to perform the behavior, goal setting, action planning, information about health consequences, and social support [5]. These BCTs appeared sufficient to support significant improvements in PA levels upon completing the 12-week intervention compared to baseline and to maintain PA behavior at the 24-week follow-up. Although additional BCTs were embedded within the mobile app (accessible to the intervention group during both the initial 12-week intervention and maintenance period), these apparently did not further improve PA levels. Evidence supporting the safety and efficacy of exercise for people with cancer is now well established [6], and as such, there is increasing focus on incorporating BCTs to support the maintenance of beneficial health behaviors post intervention. However, BCTs are not consistently embedded in PA programs for people with cancer, and reporting commonly lacks transparency, making replication difficult. In a Cochrane systematic review including 24 RCTs (comparing exercise intervention to usual care in sedentary adults), only 6 were based on a theoretical behavior change model [7]. BCT number and type in all included interventions were inconsistent, with the most common being prompting practice, providing instruction on performing the behavior, setting graded tasks, and self-monitoring (behavior and outcome). This contributed to a lack of evidence regarding the most effective BCTs for people with cancer, how many BCTs are sufficient, and whether these differ based on individual characteristics.

## Engagement With Mobile Health Apps

Over three-quarters (32/42) of participants who withdrew from the EXCEL trial within the first 12 weeks were in the intervention group; however, no withdrawals occurred between 12 and 24 weeks. This raises the question of whether the participants’ burden of using the mobile app along with participating in the EXCEL program was too high or whether participants simply felt it was not required. Findings from participant interviews will provide valuable insights into their app usage experiences and preferences. The app included several features that facilitate mobile health app use in the posttreatment setting, including having a cointervention alongside the app (telemonitoring and personalized feedback), easy navigation, being a single app housing all required information, visual graphs, and information on energy levels. Barriers to uptake associated with technical problems were addressed by troubleshooting support provided by EXCEL staff. Additional factors that may have improved uptake include app integration with PA trackers and the inclusion of relevant educational videos [8]. Time since treatment completion, as people learn to live with the consequences of their disease, has also been reported to be a barrier to the uptake of mobile health apps [9]. Almost half of the participants were within 3 years post treatment; however, the average posttreatment duration was not reported.

## Conclusions and Future Directions

In their conclusion, Ester et al [1] highlight the need for future research assessing PA maintenance beyond 6 months post program. The generalizability of these findings also needs consideration. Despite residing in rural and remote areas and not meeting PA guidelines at baseline, the EXCEL sample may not be representative of cancer survivors most in need of behavior change support. Participants were mainly female (n=174, 87%), had incomes>US $100,000 (n=72, 36%), and were well educated (n=146, 73% completed university or college or graduate school). This raises the question of how to best target our interventions and often limited resources to those who may require more PA behavior change support. Baseline screening assessments may be necessary to identify those with lower self-efficacy or perceived behavioral control [10] or a greater number of comorbidities for inclusion in future trials. As the field progresses and the focus continues shifting to support PA maintenance, researchers should aim to investigate different approaches to delivering BCTs (eg, number of BCTs, app vs incorporated into program sessions) and how this impacts PA levels—specifically with the EXCEL trial, directly comparing the app’s impact to the “exercise and educate” program. BCTs introduced in the app and the “exercise and educate” program might have been sufficient individually to support PA maintenance. The effectiveness of these approaches may also differ between participants and future research investigating characteristics of “responders” versus “nonresponders” may further guide tailored behavior change support to understand who requires what level of behavior change support and how this should be delivered.

## Abbreviations

BCT: behavior change technique
EXCEL: Exercise for Cancer to Enhance Living Well
PA: physical activity
RCT: randomized controlled trial

## Data Availability

Data sharing is not applicable to this article as no data sets were generated or analyzed during this study.
